# Evolution of resistance in paediatric patients with failure on antiretroviral therapy

**DOI:** 10.7448/IAS.15.6.18086

**Published:** 2012-11-11

**Authors:** C Orrell, J Levison, A Ciaranello, L Bekker, D Kuritzkes, K Freedberg, R Wood

**Affiliations:** 1 University of Cape Town, Desmond Tutu HIV Centre, Cape Town, South Africa; 2 Massachusetts General Hospital, Division of Infectious Diseases, Boston, USA; 3 Harvard Medical School, Boston, USA

## Abstract

**Introduction:**

HIV-1 resistance data to inform treatment sequencing are limited for children with virological failure on first- and second-line antiretroviral therapy (ART) in Sub-Saharan Africa.

**Methods:**

HIV-1-infected children aged ≤15 years were retrospectively identified from an ART cohort in Cape Town, South Africa (2003 to 2010). First-line ART was either non-nucleoside reverse transcriptase inhibitor (NNRTI) or lopinavir/ritonavir-based (with the exception of children <6 months old who received full-dose ritonavir as the sole protease inhibitor (PI) from 2004 to 2007). Second-line ART was the alternative regimen. Treatment outcomes, including virological failure, loss to care, death or remaining in care, were determined. Genotypic resistance testing was conducted on stored serum from children at first- or second-line virological failure (two consecutive HIV-1 RNA levels >1000 copies/ml). International AIDS Society criteria defined resistance mutations.

**Results:**

Of 472 children starting first-line ART, 352 (75%) remained in care, 45 (9%) were lost and 4 (1%) died on first-line treatment. Seventy-one (15%) had observed virological failure, and 37 of these children had specimens available for genotype testing. Eight children (22%) had wild-type virus, seven (19%) had thymidine analog mutations (TAMs), 24 (65%) had NNRTI resistance and two (5.4%) had multiple protease resistance (PR). Of the 78 children who received second-line ART, 54 (71%) remained in care, 6 (8%) were lost and 1 (1%) died during second-line treatment. Fifteen (20%) had observed virological failure; 13 had samples available for genotype. Three (23%) had wild-type virus, eight (62%) had TAMs, nine (69%) had NNRTI resistance, and five (38%) had multiple PI resistance all of whom had received full-dose ritonavir.

**Conclusion:**

Although virological failure was infrequent in children on first- and second-line ART, rates of observed resistance including multiple PR resistance after failure were high. Reasons for high rates of resistance include use of full-dose ritonavir and continued viremia. Wild-type virus was common, suggesting poor adherence or challenges in correct dosing. Genotype resistance testing in children with virological failure may optimize selection of subsequent regimens and inform recommendations for sequencing of existing ART.

